# Artificial Intelligence-Enabled ECG Algorithm for the Prediction of Coronary Artery Calcification

**DOI:** 10.3389/fcvm.2022.849223

**Published:** 2022-04-06

**Authors:** Changho Han, Ki-Woon Kang, Tae Young Kim, Jae-Sun Uhm, Je-Wook Park, In Hyun Jung, Minkwan Kim, SungA Bae, Hong-Seok Lim, Dukyong Yoon

**Affiliations:** ^1^Department of Biomedical Systems Informatics, Yonsei University College of Medicine, Yongin, South Korea; ^2^Division of Cardiology, College of Medicine, Heart Research Institute, Chung-Ang University Hospital, Chung-Ang University, Seoul, South Korea; ^3^BUD.on Inc., Seoul, South Korea; ^4^Division of Cardiology, Department of Internal Medicine, Yongin Severance Hospital, Yonsei University College of Medicine, Yongin, South Korea; ^5^Department of Cardiology, Ajou University School of Medicine, Suwon, South Korea; ^6^Center for Digital Health, Yongin Severance Hospital, Yonsei University Health System, Yongin, South Korea

**Keywords:** coronary artery calcium, atherosclerosis, coronary artery disease, electrocardiogram, artificial intelligence, deep neural network

## Abstract

Coronary artery calcium (CAC), which can be measured in various types of computed tomography (CT) examinations, is a hallmark of coronary artery atherosclerosis. However, despite the clinical value of CAC scores in predicting cardiovascular events, routine measurement of CAC scores is limited due to high cost, radiation exposure, and lack of widespread availability. It would be of great clinical significance if CAC could be predicted by electrocardiograms (ECGs), which are cost-effective and routinely performed during various medical checkups. We aimed to develop binary classification artificial intelligence (AI) models that predict CAC using only ECGs as input. Moreover, we aimed to address the generalizability of our model in different environments by externally validating our model on a dataset from a different institution. Among adult patients, standard 12-lead ECGs were extracted if measured within 60 days before or after the CAC scores, and labeled with the corresponding CAC scores. We constructed deep convolutional neural network models based on residual networks using only the raw waveforms of the ECGs as input, predicting CAC at different levels, namely CAC score ≥100, ≥400 and ≥1,000. Our AI models performed well in predicting CAC in the training and internal validation dataset [area under the receiver operating characteristics curve (AUROC) 0.753 ± 0.009, 0.802 ± 0.027, and 0.835 ± 0.024 for the CAC score ≥100, ≥400, and ≥1,000 model, respectively]. Our models also performed well in the external validation dataset (AUROC 0.718, 0.777 and 0.803 for the CAC score ≥100, ≥400, and ≥1,000 model, respectively), indicating that our model can generalize well to different but plausibly related populations. Model performance in terms of AUROC increased in the order of CAC score ≥100, ≥400, and ≥1,000 model, indicating that higher CAC scores might be associated with more prominent structural changes of the heart detected by the model. With our AI models, a substantial proportion of previously unrecognized CAC can be afforded with a risk stratification of CAC, enabling initiation of prophylactic therapy, and reducing the adverse consequences related to ischemic heart disease.

## Introduction

Atherosclerosis is a vascular disease in which cholesterol, fats, and other substances build up on the arterial wall, causing one's arteries to narrow and harden. When the lumen of a coronary artery is narrowed or obstructed due to atherosclerosis (with or without thrombosis), blood flow to the myocardium is impaired, leading to serious complications—namely, ischemic heart diseases (IHDs), such as angina pectoris and myocardial infarction (MI). The total number of people aged 20 and above in the United States who are affected by IHD is estimated to be 18.2 million (1). About 605,000 Americans have their first MI each year, with more than 200,000 experiencing a subsequent event (2). To prevent adverse clinical events, it is important to evaluate the cardiovascular risks of individual patients as early as possible, and actively manage major risk factors (e.g., high blood pressure, diabetes, dyslipidemia, and smoking) (3).

Coronary artery calcium (CAC) is used as a hallmark for coronary artery atherosclerosis due to its close correlation with atherosclerotic plaque formation (4). CAC score (also known as Agatston score) can be measured in diverse types of computed tomography (CT) examinations (5). CAC score results can be classified into several groups: no atherosclerosis (0 Agatston units), mild (1–99 Agatston units), moderate (100–399 Agatston units), severe (400–999 Agatston units) and very severe (1,000 or higher Agatston units) (6, 7). Its predictive value for cardiovascular events has been well-validated (8–13); in particular, compared to patients with a CAC score of zero, the hazard ratios for a coronary event were 3.61 for a CAC score of 1–100 (*p* < 0.001), and 7.73 or higher for a CAC score > 100 (*p* < 0.001) (9). Studies have shown that CAC progression can be observed in more than 20% of individuals with an initial CAC score of zero during a follow-up period of 3–6 years, and when CAC is detected, prophylactic treatments such as lifestyle modification, antihyperlipidemic drugs, or antiplatelet drugs, or additional tests such as exercise stress tests or coronary angiograms, can be performed to prevent disease progression (14–17). A well-known clinical indication for measuring CAC scores is intermediate risk based on the Framingham risk score—a sex-specific tool developed to estimate the risk of cardiovascular disease within the next 10 years (18). However, despite the clinical value of CAC scores in predicting cardiovascular events, their routine measurement is limited due to the high cost of taking a CT scan, radiation exposure, and a lack of widespread availability.

An electrocardiogram (ECG) is a highly sensitive, cost-effective, non-invasive, contrast-free, and radiation-free screening tool that is frequently measured during various health checkups. With recent advances in remote health and wearable technologies, portable ECG devices, such as smartwatches, are also becoming more widespread. These devices enable continuous monitoring of the ECG in everyday life. Furthermore, there is growing evidence that advanced artificial intelligence (AI) techniques with deep convolutional neural networks are capable of detecting subtle signals and patterns from ECGs that do not fit traditional knowledge and are unrecognizable by the human eye, enabling prediction of diseases that were previously unpredictable with ECGs (19). In a similar vein to these new findings, if CAC can be predicted using the application of AI technology to ECGs, patients measuring ECGs at various health checkups or other examinations can be afforded with an opportunistic risk stratification of a potentially existing CAC.

Thus, this study aimed to develop binary classification AI models based on deep convolutional neural networks that predict CAC at various levels (i.e., CAC scores of 100, 400, or 1,000) using only ECGs as input and then compare the performances of our AI models with that of logistic regression models built with traditional ECG features. Moreover, we aimed to address the generalizability of our models in different environments by externally validating our models on a dataset from a different institution.

## Methods

### Data Sources and Labeling

**Figure 1:**
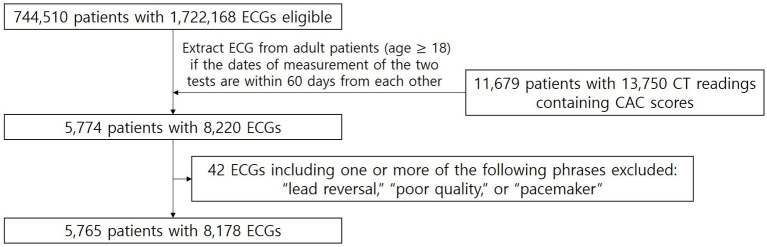
Patient flow diagram of the training and internal validation dataset from AUMC. From the AUMC ECG database, after applying the exclusion criteria, the number of ECGs measured within 60 days before or after the CT readings containing CAC scores was 8,178 ECGs from 5,765 adult patients.

For model training and internal validation, the raw waveforms of the ECGs and CAC scores were extracted from the Ajou University Medical Center (AUMC) database (Figure 1). The standard 12-lead ECG database of AUMC extracted from the General Electric Healthcare MUSE^TM^ system from June 1994 to May 2020 stored about 1.72 million units of ECG data from about 740,000 patients. The database consists of the raw waveforms; measurement data such as heart rate, PR interval, QRS duration, and QT interval; personal information, such as age, sex, height, and weight; and automatic ECG interpretations provided by built-in software. The length of each ECG is 10 s and the sampling rate is either 500 or 250 Hz. For CT readings containing CAC scores stored in the electronic medical records (EMR) database of AUMC between August 2003 (the time point when the first CT reading containing a CAC score was stored at the AUMC EMR database) and August 2018 (the last time point we were authorized to access the AUMC EMR database), we used a wide range of search terms including “agatston,” “calcium score,” “CAC score,” and “CACS” to thoroughly extract all the CAC scores stored in the database. Examples of CT readings containing CAC scores from the EMR database of AUMC are shown in Supplementary Table 1.

Among adult patients (18 years and older), the standard 12-lead ECGs were extracted if measured within 60 days before or after the CAC scores, and labeled with the corresponding CAC scores. The rationale for the time window of 60 days is that CAC is a slowly progressing disease; thus, it would be reasonable to assume that not much change can be observed in a period of a few months (15, 20). A longer time window would yield more samples to be included in our study. However, as the time window gets longer, there is a greater chance of significant change in the CAC score. Thus, we chose a time window of 60 days to balance between these trade-offs. If multiple CAC scores were measured within 60 days before or after an ECG, we labeled the ECG with the nearest measured CAC score. ECGs with automatic interpretations including one or more of the following phrases were excluded: “lead reversal,” indicating that the leads might have been misplaced; “poor quality,” indicating that the ECG contained artifacts; and “pacemaker,” indicating that an artificial pacemaker may have been present.

**Figure 2:**
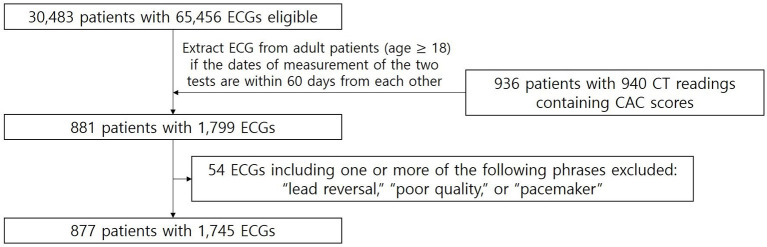
Patient flow diagram of external validation dataset from YSH. From the YSH ECG database, after applying the exclusion criteria, the number of ECGs measured within 60 days before or after the CT readings containing CAC scores was 1,745 ECGs from 877 adult patients.

The external validation dataset was extracted from the Yongin Severance Hospital (YSH) database (Figure 2). The standard 12-lead ECG database of YSH extracted from the GE Healthcare MUSE^TM^ system from March 2020 to March 2021 stored about 65,000 units of ECG data from about 30,000 patients. For CT readings containing CAC scores stored in the EMR database of YSH between March 2020 and March 2021, we used the same search terms applied to the AUMC database. Standard 12-lead ECGs were extracted if measured within 60 days before or after the CAC scores from adult patients, and labeled with the corresponding CAC scores. The length of the ECG was 10 s and the sampling rate was 500 Hz. ECGs with automatic interpretations indicating misplaced leads, poor quality, and the presence of pacemakers were excluded.

We aimed at constructing AI models predicting CAC at various levels, namely moderate CAC (score ≥ 100), severe CAC (score ≥ 400) and very severe CAC (score ≥ 1,000). Moreover, as a supplemental analysis, we constructed an AI model predicting CAC score > 0.

### Data Preprocessing and Augmentation

As the ECGs from AUMC were a mixture of 250 and 500 Hz, ECGs with a sampling rate of 250 Hz were upsampled to 500 Hz through linear interpolation. Thus, all ECGs were set to 500 Hz. We scaled the data points of each waveform to mean = 0 and standard deviation = 1 (*z*-score normalization). The formula for the *z*-score normalization would be $y_{i}^{'}=\frac{y_{i}-\mu }{\sigma }$, in which μ denotes the mean and σ denotes the standard deviation of the data point values of the original waveform. For the six limb leads (leads I, II, III, aVR, aVL, and aVF), according to the Einthoven law and Goldberger equation, the remaining four leads can be calculated if only two leads are available (21, 22). Thus, the inclusion of any two limb leads reflects no more and no less information than what can be gained from the six limb leads. Accordingly, we utilized eight leads (leads I, II, V1, V2, V3, V4, V5, and V6) from the 12 leads as input. The same preprocessing methods were applied to the external validation dataset.

We augmented our data in the training process as follows: an indiscrete 2.5 s of the 10-s ECG were randomly selected in each training epoch. Thus, a different 2.5-s interval of the ECG was selected for each training epoch. In the internal and external validation datasets, 10-s ECGs were divided into four non-overlapping segments of 2.5 s, and all segments were tested for consistency.

### Neural Network Architecture and Model Training

We constructed a deep neural network based on residual networks using only the raw waveforms of the ECGs as input (23). Supplementary Figure 1 shows the architecture of the neural network used in this study. The neural network was made up of six residual blocks. The input sequence length corresponding to 2.5 s of 500 Hz is 1,250. The number of channels for the first input was eight (number of leads used). First, the sequence length was adjusted to 1,280 by adding 15 zero paddings to each side of each channel of the input. After passing the first convolution layer with a kernel size of 15, the number of channels increased to 64, and the sequence length decreased to 640. It then passed through a batch normalization layer, rectified linear unit layer, dropout layer, and a total of six residual blocks. In each residual block, the sequence length was reduced by half. For every two residual blocks, the number of channels increased by 64, a dropout of *p* = 0.2 was applied, and the kernel size of the convolution layer decreased by 2 starting from 9. After passing through six residual blocks, the sequence length became 10 and the number of channels became 256. Then, this was flattened, passing through a fully connected layer and a softmax layer to derive the final output.

For training and internal validation, 5-fold cross-validation was performed and the average performance of each fold was derived. All cross-validation experiments (models predicting CAC score at different levels) used the same folds to enable comparison. Patients were not shared between training and validation sets. The dataset used in this study was a class imbalanced dataset and it has been established that class imbalance can have detrimental effects on classification performance, and the most widely used robust method for dealing with class imbalance is the oversampling of the minority class (24). Thus, we oversampled the minority class in the training set by simple duplication to balance each class's sample distribution as equally as possible. For example, when applied with the aforementioned data augmentation technique, if the number of samples in the majority class was about 10 times greater than that in the minority class, in each training epoch, an indiscrete 2.5 s of the 10-s ECG was randomly selected for the majority class samples, and indiscrete 2.5 s of the 10-s ECG was randomly selected 10 times for the minority class samples. The hyperparameters were tuned via extensive empirical experiments, and a learning rate of 0.001, Adam optimizer, and specific architecture of the network were chosen (Supplementary Figure 1). To prevent over-fitting we incorporated several techniques including early stopping of training based on observed validation loss, weight decay of 0.001, data augmentation (as mentioned before), and dropout layers (as mentioned before).

To address the accuracy and generalizability of a model evaluating patients from a different, but plausibly related population (25), we then performed external validation on the dataset from YSH: CAC score ≥ 100, 400, and 1,000 in the YSH dataset were validated with the corresponding models trained with all the samples in the AUMC dataset (not divided into training and internal validation datasets) with the same oversampling method and the same hyperparameters chosen in the 5-fold cross validation.

For performance comparison, we constructed logistic regression models that use traditional ECG features (Ventricular rate, PR interval, QRS duration, QT interval, QT interval corrected, P axis, R axis, T axis) provided by a built-in software from the General Electric Healthcare MUSE^TM^ system as independent variables and CAC score ≥ 100, ≥ 400, or ≥ 1,000 as dependent variables. In the AUMC dataset, the same 5-fold cross-validation approach (using the same folds) was used, and the average performance of each fold was derived. For variables with missing values, a dummy variable was created (coded 1 if the value was missing and 0 if the value was not missing). In the AUMC dataset, 3.8% of “PR interval” and 3.4% of “P axis” had missing values, and the rest had no missing values. In the YSH dataset, 8.0% of “PR interval” and 9.2% of “P axis” had missing values, and the rest had no missing values.

The average receiver operating characteristics (ROC) curve and the precision-recall (PR) curve was created for each fold of the internal validation set during 5-fold cross-validation, and the average area under the ROC curve (AUROC) and the area under the PR curve (AUPRC) of our AI model was assessed. We calculated the average accuracy, specificity, positive predictive value (PPV), negative predictive value (NPV), and F1 score at the optimal cutoff point when the Youden J statistics was at maximum for each fold (Youden J statistic = sensitivity + specificity – 1) (26). For the external validation, the ROC curves PR curves were also created and the accuracies, sensitivities, specificities, PPVs, NPVs and F1 scores were calculated at the optimal cutoff point when the Youden J statistics was at maximum based on the model trained with all the samples in the AUMC dataset.

### Statistical Analyses

The normality of continuous data was assessed with the Shapiro-Wilk test: as none of the continuous data of both institutions (AUMC and YSH) were normally distributed, they were compared by the Mann-Whitney *U*-test (two-sided). Categorical data was compared by the chi-square test. AUROCs were compared with the Delong test (27). *P*-value < 0.05 was considered as significant in all tests.

## Results

### Dataset Characteristics

Figure 1 shows the patient flow diagram of the training and internal validation dataset from AUMC. There were 1,722,168 ECGs from 744,510 patients in the source AUMC standard 12-lead ECG database from June 1994 to May 2020. The total number of CT readings with CAC scores extracted between August 2003 and August 2018 was 13,750 readings from 11,679 patients. Figure 2 shows the patient flow diagram of the external validation dataset from YSH. There were 65,456 ECGs from 30,483 patients from the source YSH standard 12-lead ECG database from March 2020 to March 2021. The total number of CT readings with CAC scores extracted between March 2020 and March 2021 was 940 readings from 936 patients.

Dataset characteristics are shown in Table 1. The AUMC dataset included 8,178 ECGs from 5,765 adult patients. The YSH external validation dataset included 1,745 ECGs from 877 patients. The AUMC dataset had significantly higher age, CAC score, and proportion of ECGs with CAC score ≥ 100, CAC score ≥ 400, and CAC score ≥ 1,000 than the YSH dataset.

**Table 1:** Dataset characteristics.

**Characteristics**	**AUMC dataset** **(*n* = 8,178)**	**YSH dataset** **(*n* = 1,745)**	***p*-value**
Number of patients	5,765	877	
Age (year)	57.17 ± 11.80	62.06 ± 13.77	<0.001
**Sex**			
Male	4,696 (57.42%)	985 (56.45%)	0.471
Female	3,482 (42.58%)	760 (43.55%)	
Average CAC score	187.82 ± 545.91	490.15 ± 1,121.55	<0.001
CAC score ≥ 100	1,836 (22.45%)	795 (45.56%)	<0.001
CAC score ≥ 400	976 (11.93%)	458 (26.25%)	<0.001
CAC score ≥ 1,000	492 (6.02%)	165 (9.46%)	<0.001

### Model Performance

**Figure 3:**
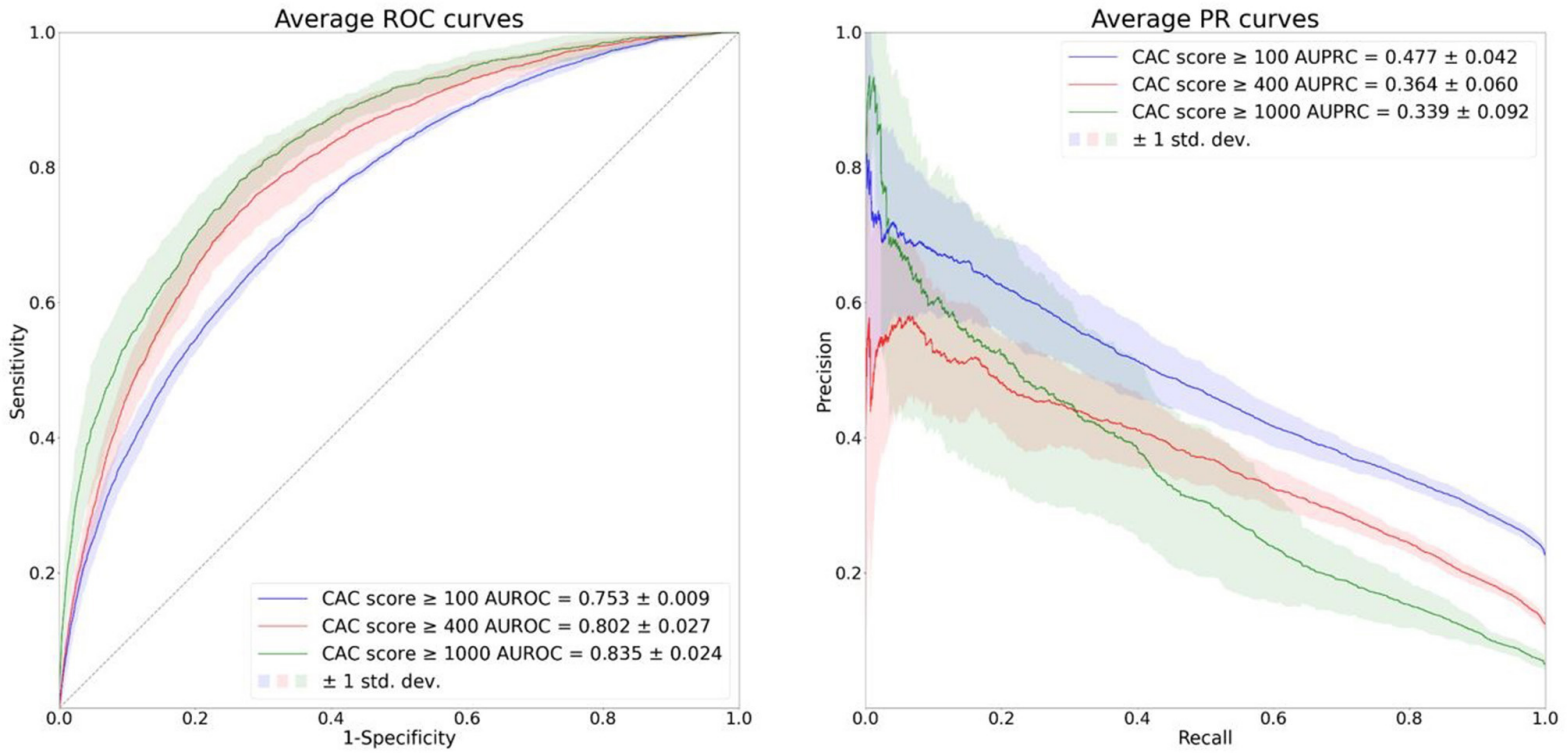
Average ROC and PR curves of the 5-fold cross validation. The solid lines depict the average ROC and PR curves and the shaded areas depict ± 1 standard deviations of the curves.

Figure 3 shows the average ROC and PR curves of the 5-fold cross validation. The AUROCs and the AUPRCs for each fold are specified in Supplementary Tables 2, 3. The average AUROCs and AUPRCs for the CAC score ≥ 100 model, CAC score ≥ 400 model and CAC score ≥ 1,000 model were 0.753 ± 0.009, 0.802 ± 0.027 and 0.835 ± 0.024, respectively and 0.477 ± 0.042, 0.364 ± 0.060 and 0.339 ± 0.092, respectively. Model performance in terms of average AUROC increased in the order of CAC score ≥ 100 model, CAC score ≥ 400 model and CAC score ≥ 1,000 model. However, model performance in terms of average AUPRC decreased in the order of CAC score ≥ 100 model, CAC score ≥ 400 model and CAC score ≥ 1,000 model, and this is due to decreasing PPV (also referred to as precision) caused by increasing class imbalance in the respective cases.

**Figure 4:**
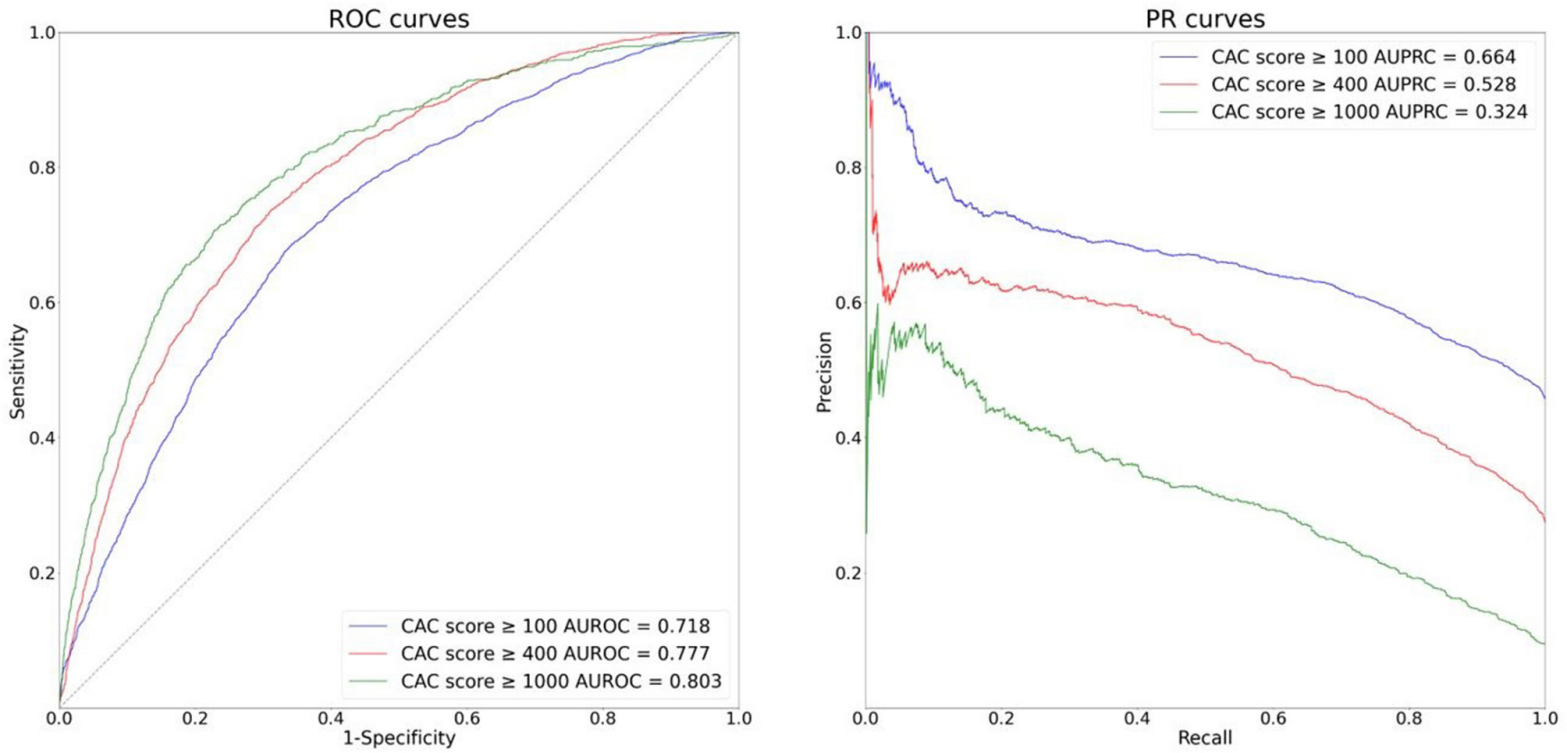
ROC and PR curves of the external validation (YSH dataset).

Figure 4 shows the ROC and PR curves for the external validation. The AUROCs and AUPRCs for the CAC score ≥ 100 model, CAC score ≥ 400 model and CAC score ≥ 1,000 model were 0.718, 0.777 and 0.803, respectively and 0.664, 0.528, and 0.324, respectively. Our models' good performance in the external validation dataset indicate they can generalize well to external environments.

Table 2 shows the performances of the models at the optimal cutoff point when the Youden J statistics was at maximum. The Youden J statistics also increased in the order of CAC score ≥ 100 model, CAC score ≥ 400 model and CAC score ≥ 1,000 model in both the cross validation and the external validation. However, the F1 score decreased in the order of CAC score ≥ 100 model, CAC score ≥ 400 model and CAC score ≥ 1,000 model; this is also due to decreasing PPV caused by increasing class imbalance in the respective cases.

**Table 2:** Performance of the models when the Youden J statistics was at the maximum.

	**Cross validation**			**External validation**		
	**CAC score ≥100**	**CAC score ≥400**	**CAC score ≥1,000**	**CAC score ≥100**	**CAC score ≥400**	**CAC score ≥1,000**
Accuracy	0.676 ± 0.031	0.744 ± 0.026	0.759 ± 0.042	0.652	0.659	0.641
Sensitivity	0.704 ± 0.046	0.732 ± 0.067	0.768 ± 0.045	0.778	0.800	0.823
Specificity	0.669 ± 0.052	0.745 ± 0.035	0.759 ± 0.047	0.546	0.609	0.622
PPV	0.384 ± 0.033	0.282 ± 0.033	0.174 ± 0.042	0.589	0.422	0.185
NPV	0.887 ± 0.013	0.955 ± 0.006	0.981 ± 0.005	0.746	0.895	0.971
F1 score	0.494 ± 0.020	0.406 ± 0.041	0.280 ± 0.055	0.671	0.552	0.302
Youden J statistics	0.373 ± 0.011	0.477 ± 0.048	0.528 ± 0.035	0.324	0.409	0.445

We report the results of the supplemental AI model predicting CAC score > 0 in Supplementary Figure 2 and Supplementary Table 4. The number of ECGs with CAC score > 0 was 3,706 (45.32%) in the AUMC dataset and 1,183 (67.79%) in the YSH dataset. In the 5-fold cross validation (AUMC dataset), the average AUROC and AUPRC were 0.696 ± 0.010 and 0.649 ± 0.024, respectively, and in the external validation (YSH dataset), the AUROC and AUPRC were 0.683 and 0.779, respectively.

Supplementary Figure 3 shows the performances of the logistic regression models. All the comparisons of the AUROCs between the ROC curves of the logistic regression models and the deep neural network models (the inference outputs of the four non-overlapping segments of 2.5 s from the 10-s ECGs were averaged to enable paired sample comparison) in each fold of the 5-fold cross validation and the external validation were statistically significant according to the Delong test, indicating that the deep neural network models using the raw ECG waveforms outperform the logistic regression models that use the traditional ECG features.

Inference outputs of all the deep neural network models are provided in Supplementary Tables 5–10. Supplementary Tables 5–9 shows the inference outputs of the deep neural network models in the validation set during the 5-fold cross validation, whereas Supplementary Table 10 shows the inference outputs of the deep neural network models in the external validation. Note that the inference outputs were derived from all the four non-overlapping segments of 2.5 s from the 10-s ECGs.

## Discussion

In this study, we developed binary classification AI models that predict CAC at various levels, using only ECGs as input. We found that our AI models performed well in predicting CAC in the training and internal validation dataset (AUROC 0.753 ± 0.009, 0.802 ± 0.027 and 0.835 ± 0.024, AURPC 0.477 ± 0.042, 0.364 ± 0.060 and 0.339 ± 0.092 for the CAC score ≥ 100 model, CAC score ≥ 400 model and CAC score ≥ 1,000 model, respectively). Our models also performed well in the external validation dataset (AUROC 0.718, 0.777 and 0.803, AUPRC 0.664, 0.528 and 0.324 for the CAC score ≥ 100 model, CAC score ≥ 400 model and CAC score ≥ 1,000 model, respectively), indicating that our model can generalize well to different but plausibly related populations. The deep neural network models using the raw ECG waveforms outperformed the logistic regression models using the traditional ECG features in all comparisons.

High NPV was observed when the Youden J statistics was at the maximum (NPV 0.887 ± 0.013, 0.955 ± 0.006, 0.981 ± 0.005 for the CAC score ≥ 100 model, CAC score ≥ 400 model and CAC score ≥ 1,000 model, respectively); this implies that our model can be a useful indicator of CAC score <100, CAC score <400 or CAC score <1,000. Although the AUROC and the Youden J statistics increased in the order of CAC score ≥ 100 model, CAC score ≥ 400 model and CAC score ≥ 1,000 model, the AUPRC and F1 score decreased, and this is due to decreasing PPV caused by increasing class imbalance in the respective cases. Thus, there is a tradeoff among performance measures (AUROC, Youden J statistics vs. AUPRC, F1 score) in our models predicting CAC score at different thresholds; further discussion would be needed to find the most appropriate compromise.

The ECG is a ubiquitous and standardized tool in clinical medicine that reflects physiological and structural condition of the heart and also give valuable diagnostic clues for systemic conditions (19). Recent AI techniques have been applied to ECGs for the automatic classification or diagnosis of various cardiac diseases, such as arrhythmia and ischemia (28–31). Moreover, there is growing evidence that advanced AI techniques with deep convolutional neural networks are capable of detecting subtle signals and patterns from ECGs that do not fit traditional knowledge and are unrecognizable by the human eye (19); for example, an AI-enabled ECG algorithm has been shown to be capable of identifying patients with atrial fibrillation during normal sinus rhythm, which has important implications for atrial fibrillation screening and the management of patients with unexplained stroke (28); another AI-enabled ECG algorithm has been shown to be capable of predicting 1-year all-cause mortality even within a subset of patients with ECGs interpreted as normal by a physician (32).

While it has been well-validated that CAC is a strong predictor of future cardiovascular events (8–13), recent studies have shown that it is associated with adverse cardiac remodeling (higher left ventricular mass and higher aortic root diameter) (33, 34). These studies show that higher CAC scores are associated with structural changes of the heart. These changes in the structure of the heart may appear as subtle changes in the ECG that were previously unrecognizable to the human eye, but can now be detected with the application of AI techniques. The fact that model performance in terms of AUROC increased in the order of CAC score ≥ 100 model, CAC score ≥ 400 model and CAC score ≥ 1,000 model in our results indicate that higher CAC scores might be associated with more prominent structural changes of the heart than the model could detect.

In a recent study by Farjo et al. (35), a logistic regression machine learning model for the prediction of CAC scores of 400 or higher using ECG features from the continuous wavelet transform and many other clinical features was built (35). To the best of our knowledge, this was the first study to develop a machine learning model predicting CAC scores. The model had an AUC of 0.868. However, none of the ECG features were included in the top three features with the highest feature importance. Instead, the top three features were coronary artery disease, age, and sex—indicating that in this study, clinical features were more important than the ECG in predicting CAC scores of 400 or higher. Although the performances of our AI model was inferior to the model constructed by Farjo et al. (35), in all aspects (for the CAC score ≥ 400 model, AUROC 0.802 ± 0.027 vs. 0.87, accuracy 0.744 ± 0.026 vs. 0.81, F1 score 0.406 ± 0.041 vs. 0.80 PPV 0.282 ± 0.033 vs. 0.79 sensitivity 0.732 ± 0.067 vs. 0.81), our study showed that CAC scores can be predicted only with ECGs, suggesting that ECGs contain enough information for this task, and that more advanced AI techniques with deep convolutional neural networks can extract complex features needed for this task.

Coronary artery atherosclerosis or calcification is a slowly progressive disease that is difficult to recognize until its symptoms develop. When the symptoms occur, there may already be serious blockages in the coronary arteries. In addition, the development of atherosclerotic plaques is a self-perpetuating cycle of oxidative stress and inflammation (36). Therefore, advanced atherosclerotic lesions are highly likely to become irreversible, and it is extremely important to detect the disease before advanced lesions appear and initiate prophylactic therapy (37). In this context, the fact that our model can predict CAC only with ECGs has important medical implications. Routine measurement of CAC scores with CT scans is limited due to high cost and concerns of radiation exposure, despite the well-established predictive value of CAC scores for cardiovascular events (8–13). In contrast, the standard 12-lead ECG is a widely used, cost-effective tool that is frequently administered during various health checkups. From the AUMC database, we found out that the total number of CAC scores measured between August 2003 (the time point when the first CAC score was stored at the AUMC EMR database) and August 2018 (the last time point we were authorized to access the AUMC EMR database) was 13,750, whereas the total number of standard 12-lead ECGs measured in the same period was 1,146,084, highly exceeding the former. Therefore, a substantial proportion of previously unrecognized CAC can be afforded with a risk stratification of CAC with our model applied on ECGs. These previously unrecognized patients, after the final diagnosis with confirmatory test, such as coronary CT scans, coronary angiographies and exercise stress tests, would be able to receive prophylactic therapy that they would not have received without our model.

Our study has numerous strengths. First, our model uses only ECGs as input and does not require additional clinical data, thus greatly enhancing real-world applicability. Second, we did not exclude ECGs based on abnormalities relating to medical conditions such as arrhythmia or ischemia. The only ECGs excluded were those with lead misplacements, unwanted artifacts, and artificial pacemakers. This means that our model is applicable regardless of cardiac conditions aside from pacemaker placement, thereby greatly enhancing the generalizability to real-world settings. Third, our model showed good external validation performances indicating that it can generalize well to foreign environments.

Our study also has some limitations. First, our model cannot be considered a confirmatory test. However, our model can help physicians recommend patients for further confirmatory testing. Physicians can incorporate the outputs of our model with other medical conditions of patients in their decision-making. Second, at present, it is difficult to explain the predictions of our model. Due to the “black box” nature of deep learning, it is currently difficult to accurately interpret which part of the ECG our model looks at to derive predictions; it may be subtle or complex ECG changes that cannot be read by the human eye. Identifying ways to overcome this “black box” nature of deep learning to enhance explainability is an ongoing area of research. Third, our model is unable to localize the branch of the coronary artery where the calcification exists. We were unable to train the model to consider the CAC score for each branch as only the “total” CAC score was recorded in the CT readings. Fourth, the performance of our AI model when applied to a truly unselected population is not known. The fact that the study population used for model training and validation in our study were prescribed CAC measurement via CTs in the first place skews them away from an unselected, general population: Some kind of clinical suspicion of a coronary artery disease might have been present before taking the CT for a considerable proportion of the study population. Our AI model would need to be trained or validated on a broader, unselected population to be potentially utilized as a true screening test. Fifth, constructing a multi-class classification model that directly predicts four classes of CAC scores (<100, ≥ 100 and <400, ≥ 400 and <1,000, and ≥ 1,000) could not be achieved due to the large increase in class imbalance when the dataset was assigned into those four classes. Future studies with much larger datasets would be required to accomplish this task.

## Conclusion

In conclusion, using only ECGs as input, we developed binary classification AI models that predict CAC at three different levels. Our models had good external validation performance, indicating that they can be generalized well to external environments. With our models, a substantial proportion of previously unrecognized CAC can be afforded with a risk stratification of CAC, enabling initiation of prophylactic therapy, and reducing the adverse consequences related to IHD.

## Data Availability Statement

The original contributions presented in the study are included in the article/Supplementary Material, further inquiries can be directed to the corresponding author/s.

## Ethics Statement

The studies involving human participants were reviewed and approved by Institutional Review Boards (IRBs) of Ajou University Medical Center (AUMC) (IRB no. AJIRB-MED-MDB-20-492) and Yongin Severance Hospital (YSH) (IRB no. 9-2021-0023). The Ethics Committee waived the requirement of written informed consent for participation.

## Author Contributions

CH and DY conceived and designed the overall study. CH extracted and analyzed the data, trained the model, interpreted the results, carried out the statistical analyses, and drafted the manuscript. DY, H-SL, and K-WK made critical revisions to the manuscript. TK developed the software for data extraction. J-SU prepared the external validation datasets. H-SL, K-WK, J-SU, J-WP, IJ, MK, and SB improved the conception and design of the study. All authors approved the final version of the manuscript.

## Funding

This work was supported by the Korea Medical Device Development Fund grant funded by the Korean government (the Ministry of Science and ICT; Ministry of Trade, Industry and Energy; Ministry of Health & Welfare; and Ministry of Food and Drug Safety) (project number 1711138152, KMDF_PR_20200901_0095). This study was also supported by a new faculty research seed money grant of Yonsei University College of Medicine for 2021 (2021-32-0044).

## Conflict of Interest

DY and TK are employees of BUD.on Inc. BUD.on Inc. did not have any role in the study design, analysis, decision to publish, or preparation of the manuscript. There are no patents, products in development, or marketed products to declare. The remaining authors declare that the research was conducted in the absence of any commercial or financial relationships that could be construed as a potential conflict of interest.

## Publisher's Note

All claims expressed in this article are solely those of the authors and do not necessarily represent those of their affiliated organizations, or those of the publisher, the editors and the reviewers. Any product that may be evaluated in this article, or claim that may be made by its manufacturer, is not guaranteed or endorsed by the publisher.
