# Supplementary material for: Artificial Intelligence-Enabled ECG Algorithm for the Prediction of Coronary Artery Calcification
Source: Front Cardiovasc Med. 2022 Apr 6;9:849223. doi: 10.3389/fcvm.2022.849223 (PMC9019148; doi:10.3389/fcvm.2022.849223)
Supplement: Supplementary file 1 [file Data_Sheet_1.DOCX]

Supplementary Material

# Supplementary Tables

Supplementary Table 1. Examples of CT readings containing CAC scores. The table shows two examples of CT readings in which regular expression was applied to extract CAC scores. We have bolded the sentences including the CAC scores.

|  | CT reading |
| --- | --- |
| Example 1 | Average heart rate : 58 bpm, Reconstruction cardiac phase : 74%  Extensive calcified plaques at all three coronary arteries.  The maximum diameter stenosis of LAD is 43%.  Moderate stenosis (57%) of 1st diagonal branch.  The maximum diameter stenosis of LCX is 39%.  Discrete near total occlusion at distal PL branch.  **Agatston calcium score is 1385.**  Global LV systolic function is within normal range.  (Indexed values, LVEF: 77%, LVEDV: 69 mL/m2)  There is no evidence of intracardiac mass in LV and LA.  Valve calcification is not seen.  No evidence of pericardial thickening or calcification or effusion.  Diffuse atherosclerosis at thoracic aorta.  Normal lung parenchyma on covered scan area.    Conclusion)  1. Extensive calcified plaques at all three coronary arteries.      - with moderate stenosis of 1st diagonal branch.      - with discrete near total occlusion at PL branch.  2. Diffuse atherosclerosis at thoracic aorta.  -- Two vessel disease  adv) coronary angiography |
| Example 2 | Average heart rate : 49 bpm, Reconstruction cardiac phase : 74%  Coronary arteries are well pacified without significant stenosis or calcified plaque.  **Agatston calcium score is zero.**  LV function is within normal range.  (LVEF: 70%, LVEDV: 86 mL)  There is no evidence of intracardiac mass in LV and LA.  Valve calcification is not seen.  No evidence of pericardial thickening or calcification or effusion.  Normal lung parenchyma and thoracic cage on covered scan area.    Conclusion)  Normal coronary CT angiogram (Ca. score=0) |

CT: computed tomography; LAD: left anterior descending artery; LCX: left circumflex artery; LVEF: left ventricular ejection fraction; LVEDV: left ventricular end-diastolic volume; LA: left atrium; LV: left ventricle.

|  | Fold 1 | Fold 2 | Fold 3 | Fold 4 | Fold 5 |
| --- | --- | --- | --- | --- | --- |
| CAC score $\geq$ 100 model | 0.740 | 0.748 | 0.755 | 0.766 | 0.755 |
| CAC score $\geq$ 400 model | 0.802 | 0.780 | 0.838 | 0.825 | 0.767 |
| CAC score $\geq$ 1000 model | 0.820 | 0.820 | 0.866 | 0.862 | 0.823 |

Supplementary Table 2. AUROCs for each fold of the five-fold cross validation using the AUMC dataset.

AUROC: area under the receiver operating characteristics curve; CAC: coronary artery calcium.

|  | Fold 1 | Fold 2 | Fold 3 | Fold 4 | Fold 5 |
| --- | --- | --- | --- | --- | --- |
| CAC score $\geq$ 100 model | 0.439 | 0.435 | 0.488 | 0.552 | 0.472 |
| CAC score $\geq$ 400 model | 0.337 | 0.315 | 0.437 | 0.434 | 0.298 |
| CAC score $\geq$ 1000 model | 0.359 | 0.193 | 0.461 | 0.284 | 0.396 |

Supplementary Table 3. AUPRCs for each fold of the five-fold cross validation using the AUMC dataset.

AUPRC: area under the precision-recall curve; CAC: coronary artery calcium.

|  | Cross validation | External validation |
| --- | --- | --- |
| Accuracy | 0.644 $\pm$ 0.013 | 0.681 |
| Sensitivity | 0.653 $\pm$ 0.066 | 0.745 |
| Specificity | 0.640 $\pm$ 0.069 | 0.546 |
| PPV | 0.603 $\pm$ 0.035 | 0.776 |
| NPV | 0.692 $\pm$ 0.032 | 0.504 |
| F1 score | 0.623 $\pm$ 0.018 | 0.760 |
| Youden J statistics | 0.293 $\pm$ 0.019 | 0.291 |

Supplementary Table 4. Performance of the CAC > 0 model when the Youden J statistics was at the maximum.

CAC: coronary artery calcium; NPV: negative predictive value; PPV: positive predictive value.

# Supplementary Figures


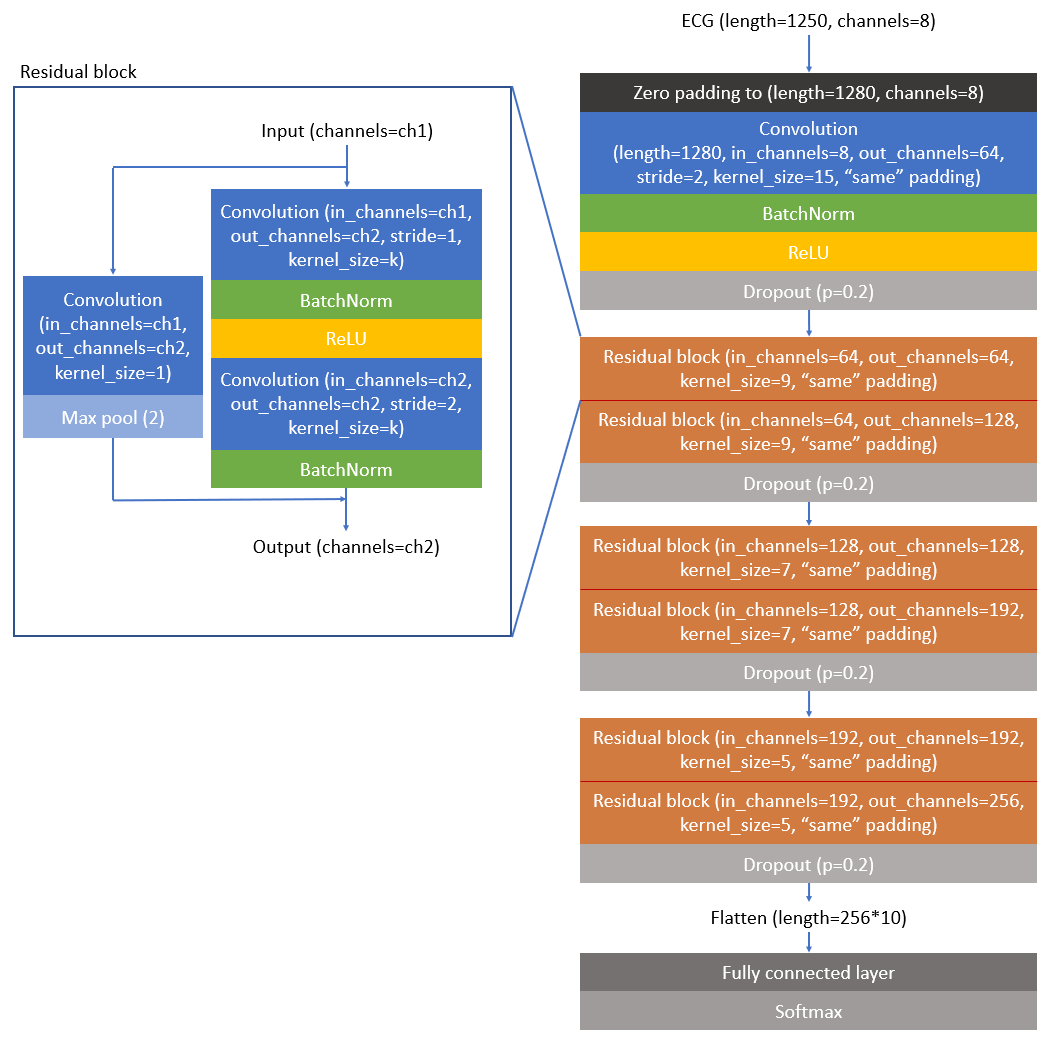


Supplementary Figure 1. Neural network architecture for the prediction of coronary artery calcium (CAC) score. The neural network is made up of six residual blocks. The specific architecture of the network was tuned via various empirical experiments.

ECG: electrocardiograms


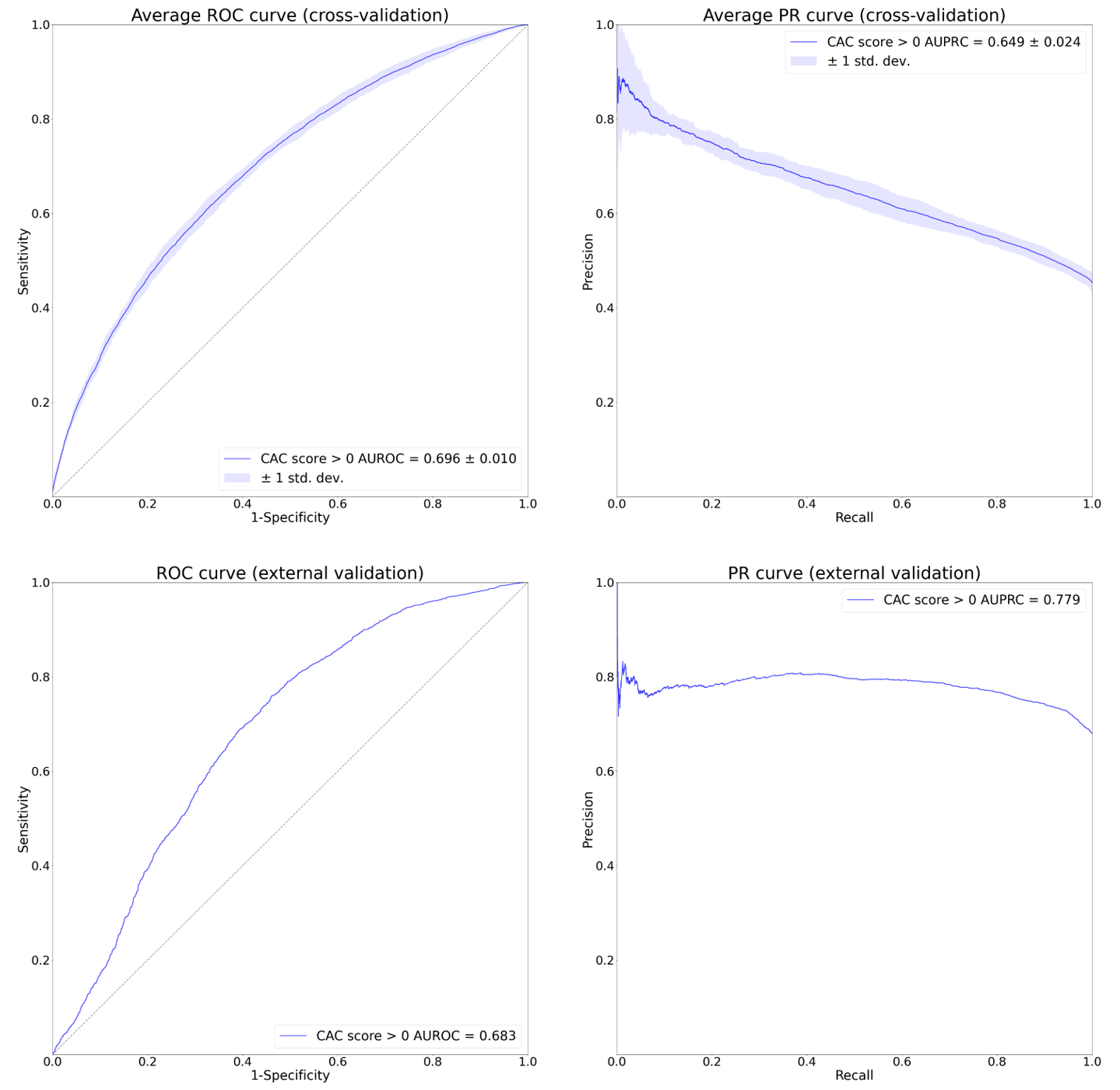


Supplementary Figure 2. ROC and PR curves of the CAC > 0 model (deep neural network).

AUPRC: area under the precision-recall curve; CAC: coronary artery calcium; ROC: receiver operating characteristics; PR: precision-recall.


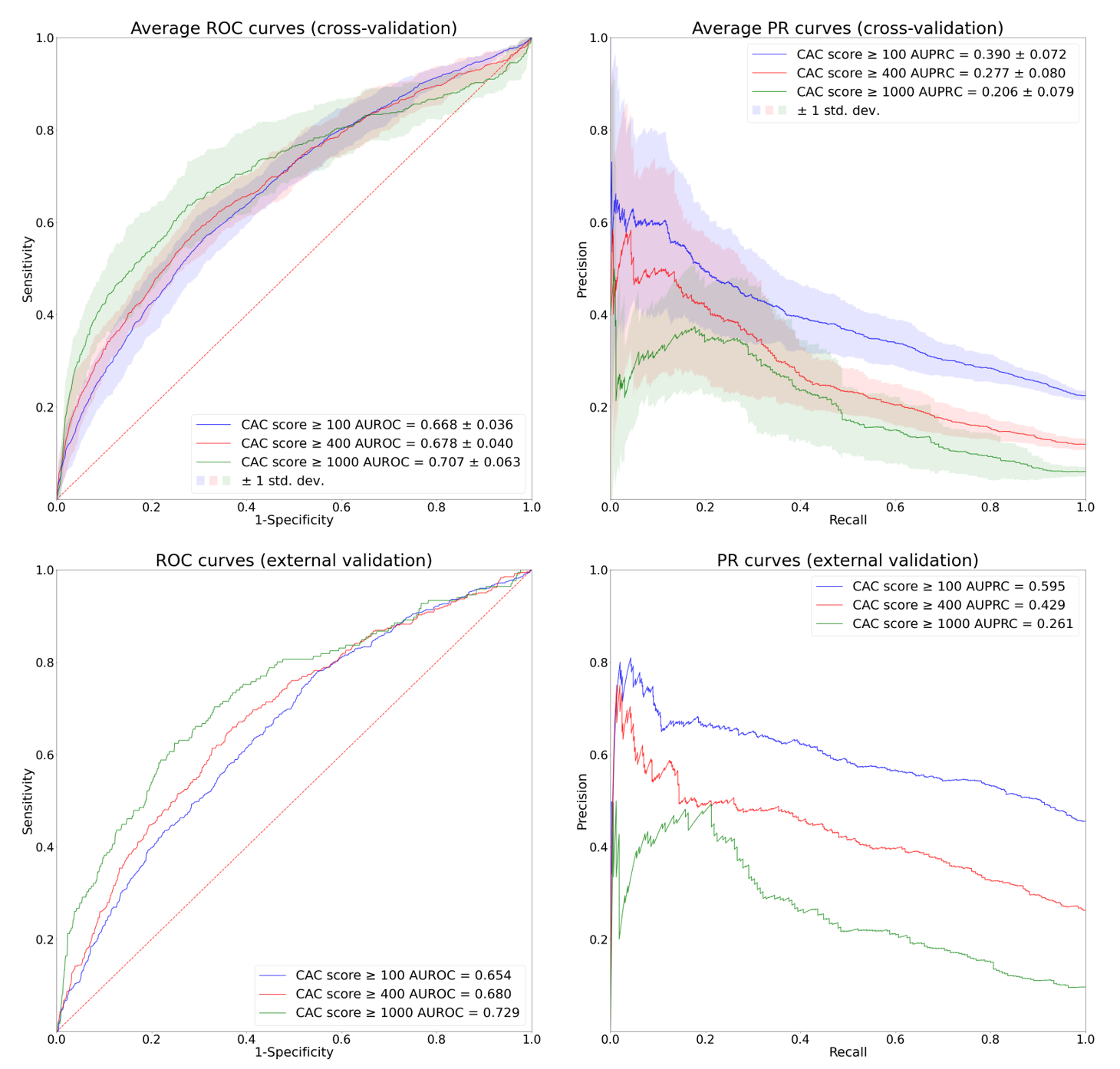


Supplementary Figure 3. ROC and PR curves of the logistic regression models

AUPRC: area under the precision-recall curve; CAC: coronary artery calcium; ROC: receiver operating characteristics; PR: precision-recall
